# Onchocerciasis transmission in Ghana: biting and parous rates of host-seeking sibling species of the *Simulium damnosum* complex

**DOI:** 10.1186/s13071-014-0511-9

**Published:** 2014-11-21

**Authors:** Poppy HL Lamberton, Robert A Cheke, Martin Walker, Peter Winskill, Mike Y Osei-Atweneboana, Iñaki Tirados, Anthony Tetteh-Kumah, Daniel A Boakye, Michael D Wilson, Rory J Post, María-Gloria Basáñez

**Affiliations:** London Centre for Neglected Tropical Disease Research, Department of Infectious Disease Epidemiology, School of Public Health, Imperial College London, St Mary’s Campus, London, W2 1PG UK; Natural Resources Institute, University of Greenwich at Medway, Central Avenue, Chatham Maritime, Kent, ME4 4TB UK; MRC Centre for Outbreak Investigation and Modelling, Department of Infectious Disease Epidemiology, School of Public Health, Imperial College London, St Mary’s Campus, London, W2 1PG UK; Department of Environmental Biology and Health, Council for Scientific and Industrial Research, Water Research Institute, Accra, Accra, PO Box M32, Ghana; Department of Vector Biology, Liverpool School of Tropical Medicine, Pembroke Place, Liverpool, L3 5QA UK; Ghana Health Service, Private Mail Bag, Ministries, Accra, Ghana; Noguchi Memorial Institute for Medical Research, University of Ghana, Legon, Accra, PO Box LG581, Ghana; School of Natural Sciences and Psychology, Liverpool John Moores University, Byrom Street, Liverpool, L3 3AH UK; Disease Control Department, London School of Hygiene and Tropical Medicine, Keppel Street, London, WC1E 7HT UK

**Keywords:** *Simulium damnosum* s.l, *S. damnosum* s.s, *S. sirbanum*, *S. squamosum* form C, *S. squamosum* form E, Beffa form of *S. soubrense*, *S. yahense*, *S. sanctipauli*, Biting rate, Parous rate, Host-seeking behaviour

## Abstract

**Background:**

Ghana is renowned for its sibling species diversity of the *Simulium damnosum* complex, vectors of *Onchocerca volvulus*. Detailed entomological knowledge becomes a priority as onchocerciasis control policy has shifted from morbidity reduction to elimination of infection. To date, understanding of transmission dynamics of *O. volvulus* has been mainly based on *S. damnosum sensu stricto* (s.s.) data. We aim to elucidate bionomic features of vector species of importance for onchocerciasis elimination efforts.

**Methods:**

We collected *S. damnosum sensu lato* from seven villages in four Ghanaian regions between 2009 and 2011, using standard vector collection, and human- and cattle-baited tents. Taxa were identified using morphological and molecular techniques. Monthly biting rates (MBR), parous rates and monthly parous biting rates (MPBR) are reported by locality, season, trapping method and hour of collection for each species.

**Results:**

*S. damnosum* s.s./*S. sirbanum* were collected at Asubende and Agborlekame, both savannah villages. A range of species was caught in the Volta region (forest-savannah mosaic) and Gyankobaa (forest), with *S. squamosum* or *S. sanctipauli* being the predominant species, respectively. In Bosomase (southern forest region) only *S. sanctipauli* was collected in the 2009 wet season, but in the 2010 dry season *S. yahense* was also caught. MBRs ranged from 714 bites/person/month at Agborlekame (100% *S. damnosum* s.s.*/S. sirbanum*) to 8,586 bites/person/month at Pillar 83/Djodji (98.5% *S. squamosum*). MBRs were higher in the wet season. In contrast, parous rates were higher in the dry season (41.8% vs. 18.4%), resulting in higher MPBRs in the dry season. Daily host-seeking activity of *S. damnosum* s.s.*/S. sirbanum* was bimodal, whilst *S. squamosum* and *S. sanctipauli* had unimodal afternoon peaks.

**Conclusions:**

The bionomic differences between sibling species of the *S. damnosum* complex need to be taken into account when designing entomological monitoring protocols for interventions and parameterising mathematical models for onchocerciasis control and elimination.

**Electronic supplementary material:**

The online version of this article (doi:10.1186/s13071-014-0511-9) contains supplementary material, which is available to authorized users.

## Background

Ghana has a great diversity of sibling species of the *Simulium damnosum* complex [1], vectors of *Onchocerca volvulus,* the causative agent of human onchocerciasis or river blindness [2]. Sibling species and cytoforms, of which there are approximately 60 [3,4], differ in their geographical distribution, ecology, degree of anthropophagy and vectorial capacity [3,5]. Historically, species identification has been based on cytotaxonomy of the differences in polytene chromosomes of blackfly larvae [6,7], collected from breeding sites [8]. Adult populations of the important West African sibling species found in Ghana—*S. damnosum sensu stricto* (s.s.) Vajime and Dunbar, *S. sirbanum* Vajime and Dunbar, *S. sanctipauli* Vajime and Dunbar, *S. yahense* Vajime and Dunbar, *S. soubrense* Beffa form [9], and *S. squamosum* Enderlein (of which both C and E forms occur) —can be distinguished to morphospecies [5,10], and individuals of *S. yahense* can be identified [11]. However, *S. squamosum* shares many morphological traits with other sympatric species, causing difficulties when identifying some adult blackflies [10]. Thus, there is a paucity of information on host-seeking behaviour and spatio-temporal distributions of the various taxa, complicating assessment of their vectorial role and inclusion in *O. volvulus* transmission dynamics models. However, almost all adult blackflies from Ghana can now be fully distinguished to species using a combination of morphological [5,12–15] and molecular techniques, with *S. squamosum* lacking a 10-base pair indel from the non-transcribed H3-H4 histone intergenic spacer region [16].

Detailed historical knowledge exists of the geographical distribution of vectors in southern Ghana, updated by larval samples collected during this study [1]. In brief, *S. damnosum* s.s. and *S. sirbanum* are found in the savannah areas of the north-centre, *S. sanctipauli* and *S. yahense* in the southern forested regions, and *S. soubrense* and *S. squamosum* (form C east of the Volta Lake and form E west of it), across the forest and savannah mosaic. However, knowledge of spatial and temporal host-seeking patterns, and of biting and parous rates of the different taxa, remains to be fully elucidated in many African countries. Detailed entomological understanding has been recognised as a priority as the focus of onchocerciasis interventions has shifted from morbidity control to elimination of the infection where deemed possible [17]. Models of *O. volvulus* transmission dynamics that investigate intervention outcomes are mostly parameterised using *S. damnosum* s.s.*/S. sirbanum* [18–26], with the exception of the model by Davies (1993) [27], based on transmission of forest onchocerciasis by *S. soubrense* B *sensu* Post, some quantitative analyses on other *S. damnosum* complex species including *S. leonense* and *S. squamosum* B [28,29], and a recent modelling study of the effect of climate change on *O. volvulus* transmission in Ghana and Liberia, including *S. soubrense* [30]. This research gap will need addressing in areas where different species compositions exist in the human-biting blackfly population. Since species with varying vector competence may respond differently to reduced microfilarial load, this becomes particularly important as programmes progress towards elimination. In these instances, deployment of intervention tools not only reliant on mass administration of ivermectin may need to be considered.

In Neotropical America, knowledge of adult species distributions, biting rates and parous rates recently increased [31–34], motivated by the necessity of the Onchocerciasis Elimination Program for the Americas (OEPA) to understand where and when to conduct entomological monitoring to evaluate elimination efforts. Similarly detailed data on vector distribution and bionomics will be crucial for understanding the epidemiology of infection and transmission patterns in parts of Africa shifting towards elimination goals [17]. Diurnal and locality-specific variations in simuliid biting densities have been associated with variations in air temperature, relative humidity, and river levels as well as with forest versus savannah ecology [32–37].

We embarked on a comprehensive entomological study, during both wet and dry seasons, at a range of sites in Ghana that had been under vector control during the Onchocerciasis Control Programme in West Africa (OCP) and/or are currently under ivermectin-based control. In this paper we present data on adult blackfly species composition, vector abundance, host-seeking behaviour and physiological age by locality, season and collection method. We discuss the possible epidemiological implications of our results and the need to include ensembles of relevant vector species in population dynamics models for investigation of the transmission, control and elimination of onchocerciasis in African settings.

## Methods

### Ethical statement

Ethical clearance was obtained from the Imperial College Research Ethics Committee (ICREC_9_1_7) and the Institutional Review Board of the Noguchi Memorial Institute for Medical Research, University of Ghana (IRB:0001276, 006/08-09). No tissue samples were taken from human subjects; however, local villagers and elders assisted with blackfly collections. Signed informed consent was obtained from all individuals involved after detailed explanations in their local languages about the study. Participating individuals were not at an increased risk of exposure, nor were human samples obtained for diagnosis, therefore no treatments were offered. However, all participants were receiving ivermectin as part of the national programme following appropriate (annual or biannual) schedules according to the Ghana Health Service strategy [38].

### Study area

**Figure 1:**
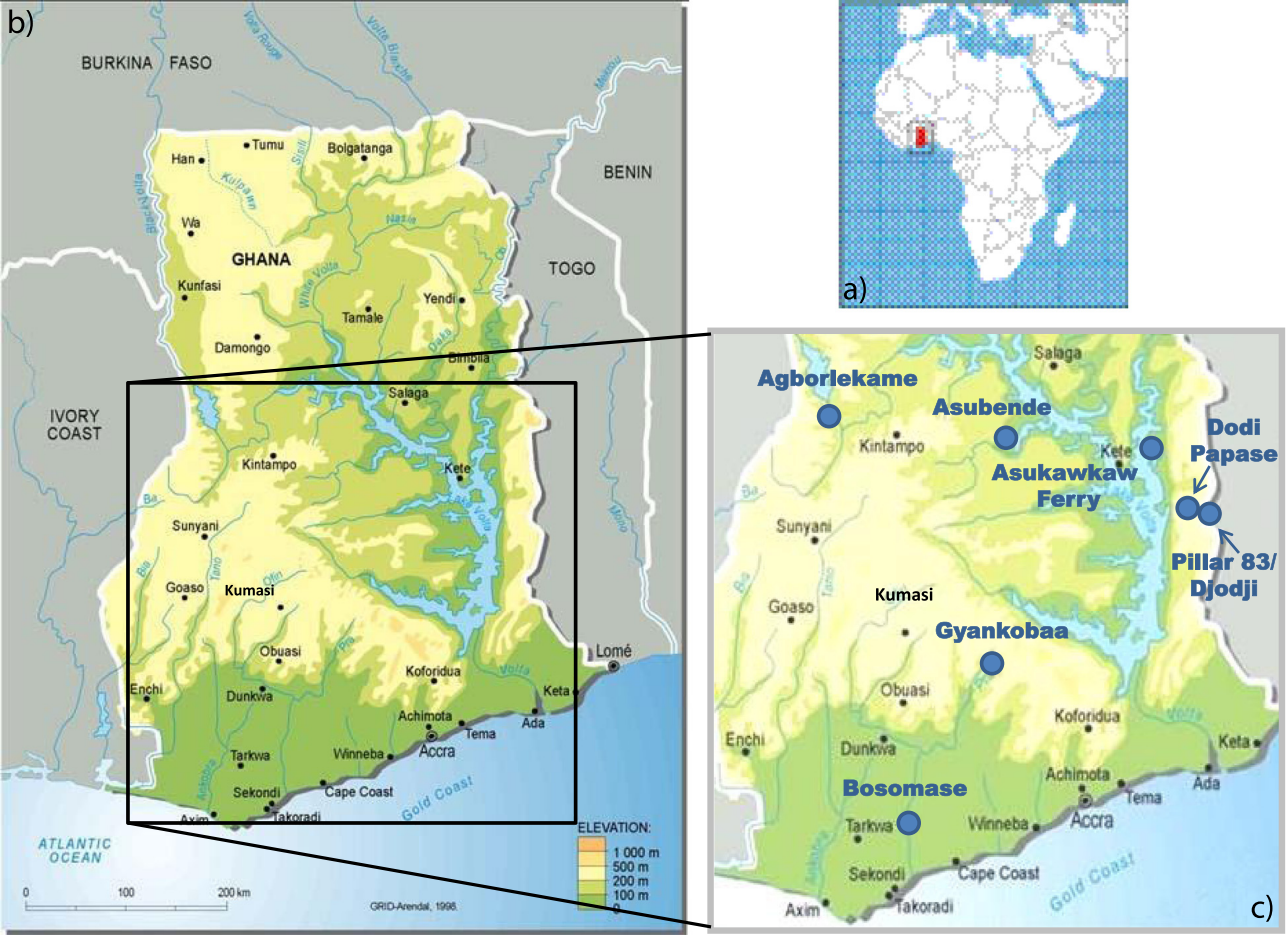
**Maps showing the location of Ghana (a) and the seven Ghanaian study sites (b and c).**

Study sites were selected according to vector species, bioclimatic zone, vector control, ivermectin distribution history, and variability in village size and domestic animal populations (for a census of possible blood hosts, the results of which will be presented elsewhere), during a preliminary survey conducted between 27^th^ April and 14^th^ May 2009, when blackfly larvae were also collected from breeding sites for cytotaxonomic identification [1]. Subsequently, the main field work was conducted during one wet season, 23^rd^ July–5^th^ September 2009, and two dry seasons, 14^th^ February–28^th^ March 2010 and 30^th^ January–5^th^ March 2011. Not all sites were successfully sampled during each period due to weather conditions and variability in blackfly population abundance. Blackfly collection was conducted in seven villages within four regions of Ghana (Figure 1), namely:

### Brong-Ahafo region

***Asubende*** (08°01'01.4"N, 00°58'53.8"W), situated on the river Pru in Guinea savannah woodland; the banks of the river are partially vegetated with trees and surrounded mainly by land cleared for agriculture. The main onchocerciasis vector is *S. damnosum* s.s., but *S. sirbanum* is also found in this area [39]. Vector control started in January 1986, but was interrupted several times during 1987–1989 because of ongoing trials of the impact of ivermectin mass treatment on transmission [39,40]. This site has been used in a series of onchocerciasis studies [38–42], partly due to its high baseline microfilarial load and prevalence [39,40], and it is the village whose data have been used to parameterise ONCHOSIM [18]. Annual ivermectin distribution commenced in 1987 and biannual distribution started in 2009.

***Agborlekame*** (08°14'04.0"N, 2°12'23.2"W), located on the Black Volta River and with similar vegetation to Asubende; however, the site was within sight of a dam being constructed upstream in the Bui Gorge, which was completed in late 2013; hence there will be major ecological and epidemiological changes associated with this site in the future. The only species recorded at the site during this study were *S. damnosum* s.s./*S. sirbanum*. Agborlekame was also included in the OCP from Phase I, with vector control from 1975, and annual ivermectin distribution from 1988. Biannual distribution was initiated in 2009.

### Volta region

***Asukawkaw Ferry*** (07°40'55.9"N, 00°22'16.0"E), positioned on the edge of Asukawkaw village on the river Asukawkaw, was an OCP vector capture site and under experimental vector control in 1981 [43], and received routine larviciding from February 1988 until the end of the OCP. It adjoins the Asukawkaw Forest Reserve, but a few kilometres downstream of Asukawkaw the river is surrounded by savannah and agricultural land, before draining into the Volta Lake. The main vector taxon present here is *S. squamosum* C which occurs sympatrically with a few *S. damnosum* s.s. and the Beffa form of *S. soubrense* [44]. Before its eradication [45], the Djodji form of *S. sanctipauli* was very common at this site [46]. Annual ivermectin distribution commenced in 1993; the biannual ivermectin strategy has not been adopted.

***Dodi Papase*** (07°43'22.5"N, 00°30'38.3"E), on the river Wawa, a tributary of the Asukawkaw river, is situated in a well wooded, formerly well forested, zone in the Volta region highlands. However, like many sites close to communities of substantial sizes it is subject to much deforestation. The main vector taxa here are *S. squamosum* C with a few *S. damnosum* s.s. [1]. Historically the Djodji form of *S. sanctipauli* was common at this site. Annual ivermectin distribution commenced in 1993; the biannual ivermectin strategy has not been adopted.

***Pillar 83*** (07°42'20.3"N, 00°35'21.5"E), Ghanaian village on the border with Togo, where the breeding site was known as **Djodji** in the OCP studies. This site, on the Wawa river (known as the Gban-Houa river in Togo), is the type locality of the Djodji form of *S. sanctipauli*, now extinct, which used to be very common together with *S. squamosum* C [47], and there have been isolated records of *S. damnosum* s.s., *S. sirbanum* and *S. yahense* [1]. Annual ivermectin distribution commenced in 1993; the biannual ivermectin strategy has not been adopted.

The breeding sites at Asukawkaw Ferry, Dodi Papase and Pillar 83 were first treated with larviciding insecticides during OCP experimental campaigns (reinvasion studies) in 1981 (see Figure two of Cheke & Garms [43]), before becoming part of the South-eastern extension zone which reached these river basins when it became fully operational in February 1988.

**Figure 2:**
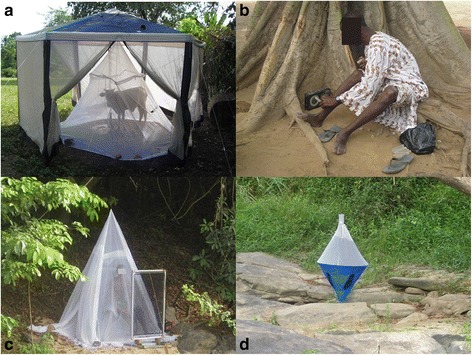
**Methods used to collect host-seeking female blackflies.** Methods included **(a)** a cow, protected by a mosquito net, inside a tent (Cow-baited tent); **(b)** the standard OCP vector collection method; **(c)** electric nets near a human protected by a mosquito net, and **(d)** bi-conical traps baited with human odour.

### Western region

***Bosomase*** (05°10'44.7"N, 01°36'23.1"W) is a forested site on the river Pra where the river is over 30 metres wide, making it a typical habitat for *S. sanctipauli*. However, since this site was not an OCP vector prospection/capture point, no records of cytotaxonomic blackfly identifications were available to the authors prior to this study. Bosomase was not part of the OCP but was incorporated into the national programme in 2003, when it began receiving annual community ivermectin treatment.

### Ashanti region

***Gyankobaa*** (06°20'12.4"N, 01°16'11.3"W), located on the river Anum in forest; *S. sanctipauli* and *S. damnosum* s.s. have been reported here commonly with several additional records of *S. squamosum* E, *S. yahense* and *S. sirbanum* [1]. Gyankobaa was not part of the OCP and therefore was not subjected to vector control but was incorporated into the National Onchocerciasis Control Programme in 2006, receiving biannual ivermectin distribution from 2010.

### Blackfly sample collection

#### Host-seeking blackflies

##### Human- and cattle-baited tents

Two hexagonal navy blue cloth (gazebo-type) tents, of dimensions 4 m × 4 m × 2.6 m high, with white mosquito mesh walls were positioned at similar distances from the river (one baited with a person (males aged 18 to 45 years) and the other with a bovine calf (Figure 2a)), separated by at least 20 metres. A mosquito net protected the human or calf from blackfly bites yet permitted collection of host-seeking flies attracted to the host on the tent walls and ceiling. Each tent and mosquito net was specifically allocated to either human or cattle, to prevent cross contamination of olfactory attractants, and was washed in water without detergent after each catching session. The human and cow attractants were assigned at random, and positions were reversed after the first two of four collection days. Two sides of the tents were open to allow simuliids to fly in. As blackflies fly towards light, sides in the sunshine were kept shut to maximise trapping of flies after being attracted to, but unsuccessfully biting on, the protected host. Traps were set from 7 am to 6 pm daily for four days at each site and sampling period, and after the first 50 minutes of each hour all entrances were closed and flies collected. The calf was fed and watered *ad libitum* and the human swapped at lunchtime. Human attractants were swapped such that they did not work during the same time slot on consecutive days. Blackflies inside the tents were collected with aspirators at hourly intervals, and ambient temperature and relative humidity recorded. Any notable strong winds or precipitation were recorded [36]. Flies were stored alive in plastic tubes in a cool box during the day and transferred to a refrigerator in the evening for subsequent identification and dissection the following day. (The motivation for collecting flies attracted to cattle stemmed from the objective of the overall study, aiming to understand patterns of blood host choice by onchocerciasis vectors and their impact on transmission dynamics. Such patterns will be reported elsewhere).

##### Vector collectors

Vector collectors were not used in the three Volta Region villages of Asukawkaw Ferry, Dodi Papase and Pillar 83, at the start of the study in the wet season of 2009 as this was not part of the original protocol, which intended to replace vector collectors by the host-baited tents. However, collection protocols were revised as it was soon realised that human-baited tent results did not accurately reflect catching rates previously recorded by OCP vector collectors. Therefore, standard OCP vector collector techniques from 7 am to 6 pm [48], for up to five consecutive days, were adopted for all subsequent sampling (Figure 2b) (Table 1) to estimate the daily landing rate (a proxy for vector biting rate). When possible, the vector collectors were positioned at historical OCP sites enabling direct comparisons with OCP vector capture databases. Collectors wore shirts and trousers rolled up to the knee and constantly watched their lower legs for landing blackflies which were caught individually, before procuring a blood meal, and trapped in polypropylene tubes (10 cm × 1.5 cm). Flies were stored at hourly intervals in a cool box as above for subsequent identification and dissection.

**Table 1:** **Summary of blackfly collection per region, village, season and trapping technique**

**Region**	**Village**	**2009 wet season**	**2010 dry season**	**2011 dry season**
**Brong-Ahafo**	Asubende	-	-	Human, Cow, V/C
	Agborlekame	-	Human, Cow, V/C	-
**Volta**	Asukawkaw Ferry	Human^a^, Cow^b^	Human, Cow, V/C	Human, Cow, V/C
	Dodi Papase	Human, Cow	Human, Cow, V/C	Human, Cow, V/C
	Pillar 83/Djodji	Human, Cow	Human, Cow, V/C	Human, Cow, V/C
**Western**	Bosomase	Human, Cow, V/C^c^	Human, Cow, V/C	-
**Ashanti**	Gyankobaa	Human, Cow, V/C	-	-

^a^Human: flies collected from the human-baited tent; ^b^Cow: flies collected from the cattle-baited tent; ^c^V/C: flies collected by the standard OCP vector collection method.

#### Other host-seeking capture methods

##### Electric nets

Preliminary tests with electric nets, successfully used for tsetse flies *Glossina* spp. [49–51] and mosquito [52] collections, were trialled as potential replacements of vector collector methods. These traps were used for blackfly collection on sporadic dry days during the rainy season in 2009 and followed up with a full feasibility investigation during a two-week period in August 2010 in the dry season. Nets, of 1 m × 0.5 m, were placed along the river banks at Agborlekame, Bosomase and Gyankobaa either alone, in the presence of a human attractor (protected by a mosquito net) (Figure 2c) or with a human-odour-baited black cotton sheet placed within the net.

##### Biconical traps

Challier-Lavéissière biconical (tsetse) traps (Figure 2d), modified with mesh in the catching chambers sufficiently small to prevent blackflies from escaping, were tested over a two-week period during August 2010 at Agborlekame, Bosomase and Gyankobaa (as they had shown promise with *S. damnosum sensu lato* (s.l.) in earlier trials) [53,54]. They were set on their own (clean or baited with human odour from a man sleeping on the net the night before) and in the presence of a human attractor (protected by a net) sitting nearby during the day. Flies were collected in the evening from both traps and identified to species.

#### Blackfly species identification and parous rates

Hourly-caught flies from human-baited tents, cattle-baited tents and vector collectors were dissected the following day. If overall numbers were too great (>300 per day) sub-samples of five flies per hourly slot (per catching technique per day) were dissected. Each fly was individually anaesthetized using chloroform or ethyl acetate, placed on a microscope slide with a drop of saline solution and morphologically identified by scoring the colour of wing tufts [14], post-cranial hairs [5,15], and ninth tergite hairs [13], and by measuring the thorax/antenna ratio [12,13]. The colour of the fore-coxae used by some authors [10,55] to separate *S. damnosum* s.s. from *S. sirbanum* is unreliable since many individuals of both species and of *S. squamosum* with either dark or pale fore-coxae have been noted, especially in the eastern parts of the former OCP (R. Garms & RAC, unpublished data), and therefore this character was not used for species identification, although it helped to separate members of the *S. sanctipauli* sub-complex and *S. yahense* from the savannah species. After morphological identification the flies were dissected for parity status by the presence or absence of ovarian relics [56] by RAC. Nulliparous females were pooled in absolute ethanol for each host attractant at each location for future analysis. Parous females’ abdomens were separated from the head and thorax, which were preserved individually in correlating wells of two 96-well PCR plates (one for heads plus thoraces, one for abdomens) in absolute ethanol. The abdomens were used for molecular identification of *S. squamosum* [16]. Morphological and molecular results were combined for final species identification, with molecular results taken as definitive. When any conflicting results arose, blackfly larval identifications [1] and molecular results from all flies collected, with all collection methods, were used to inform the identifications by providing locality-specific cut offs for the thorax/antennae ratios, which were adjusted on a village and season basis, for final species identification.

*Simulium damnosum* s.s. and *S. sirbanum* are often sympatric and morphologically similar; therefore, due to uncertainties in their definitive identification, with previous molecular techniques not proving to be robust, results for these species were combined. Any remaining undissected flies were morphologically identified in the laboratory in the UK and head plus thoraces and abdomens separated and stored as described above.

### Data analysis

This paper focuses on the numbers of each simuliid species, expressed as the monthly biting rates (MBRs), caught in each locality and season, the percentages of parous flies, and the monthly parous biting rates (MBPR) summarising the density of host-seeking flies that constitute the epidemiologically important section of the biting population. When fly numbers were greater than approximately 300 flies caught per day, priority for parous rate dissections was given to blackflies caught in human- and cow-baited tents over vector collections, minimising the risk that blood meals taken during collection could bias molecular identification of the previous blood hosts. Statistical analyses were carried out using SPSS version 21 (SPSS, Inc., Chicago, IL, USA) or R [57].

#### Biting and parous rates

Daily biting rates (DBRs) were calculated as the total number of flies caught over 11 hours, from 7 am until 6 pm, as per OCP protocols [48]. When the full 11 hours were not sampled, we calculated the arithmetic mean hourly biting rate from all days from the available data and multiplied this by 11 to estimate the DBR. Monthly biting rates (MBRs) were calculated in strata defined by sibling species (but also for overall *S. damnosum* s.l.), location, season and trapping method. These were calculated by multiplying the stratum-specific (defined by species, location and season) total number of each species caught in a month by a constant defined by the number of sampling hours completed and the number of (11-hour) days in the month in question. Data were not collected throughout the whole year so we did not calculate annual biting rates. Paired *t*-tests were used to determine whether differences in mean hourly catching rate for each hour of each day within the same stratum, estimated from data collected from vector collectors and human-baited tents or cattle-baited tents, were statistically significant. Freidman tests were performed to investigate the relative catching success across all three catching techniques at each village. A paired *t*-test was used to determine whether differences in the hourly numbers of “non-*damnosum*” simuliids (i.e. not members of the *S. damnosum* complex), caught by man- and cow-baited tents were statistically significant. Differences in the numbers of blackflies of different species caught in rainy and dry seasons within each locality were assessed for statistical significance by chi-squared tests. Host choice was compared using chi-squared tests on the numbers of blackflies caught in the man- or cow-baited tents at each locality and season for each taxon. The statistical significance of differences in biting rates between seasons was determined using (non-paired) *t*-tests.

The parous rate was defined as the percentage of parous females in a sample of dissected female flies in strata defined by taxon, location, season and trapping method over all sampling hours and days. Exact 95% binomial confidence intervals (CI) were determined using the method of Clopper-Pearson [58]. Differences in parous rates between dry and wet seasons were assessed for statistical significance using (non-paired) *t*-tests, invoking the asymptotic normal approximation to the binomial distribution.

The monthly parous biting rate (MPBR) was calculated by multiplying the parous rate by the MBR for each species, location, season and trapping method. Uncertainty in MPBRs was driven by uncertainty in the estimated parous rate as opposed to the MBR which was calculated from the total number of flies caught in a month multiplied in a manner reflecting the hours of sampling and the days in the month, as described above.

Of the 9,916 flies collected, 996 were not fully identified to species even after using both morphological and molecular techniques (Additional file 1). It was assumed that non-identified flies represented a random subsection of the total caught. Therefore, when necessary, MBRs and MPBRs for each species were estimated using the proportions of the fully identified flies extrapolated up to the total numbers.

#### Diurnal distribution of biting and parous rates

The diurnal distribution of blackfly biting was assessed using the raw data on the number of blackflies caught by vector collectors in each hour-long sampling interval, viz. hourly biting rates (HBRs). Arithmetic mean HBRs were estimated in strata defined by sibling species and season, but pooled by location (village). Combining data from different locations risked introducing, or exacerbating existing, extra-Poisson variation (overdispersion) in HBRs. Hence, it was assumed that variation in HBRs was inflated relative to the Poisson variance (i.e. the mean) by a multiplicative constant, or dispersion parameter. This ultimately led to more realistic, albeit conservative, estimates of uncertainty (standard errors, SEs) and confidence intervals. Standard errors were calculated from the estimated dispersion parameter, the arithmetic mean and the stratum-specific sample size. In turn, these SEs were used to construct approximate (asymmetric) confidence intervals around the mean HBR, initially on a logarithmic scale, and then after back-transformation, on the original scale of the counts (HBRs). This approach is used frequently for constructing approximate confidence intervals for overdispersed Poisson data particularly in conjunction with generalized linear model (GLM) analyses, or more specifically quasi-Poisson models [59].

A similar approach was taken to assess the diurnal distribution of blackfly parity. However, in contrast to the analysis of diurnal biting rates which used vector collector data only, and because not all of the blackflies caught were dissected for parity, data from the vector-collectors, man- and cow-baited traps were first pooled, before being further combined over location (village). Like the mean HBRs, this process of pooling data over variables potentially introduces or exacerbates existing extra-binomial variation (overdispersion). Hence, and by analogy with estimation of SEs for the mean HBRs using a quasi-binomial approach, the variance of the number of parous flies identified per hour was assumed to be inflated relative to the binomial variance by a multiplicative dispersion constant [59]. Confidence intervals were then constructed using this inflated SE, first on a logarithmic (logit) scale, and then after back-transformation, on the original scale of the proportion.

## Results

A total of 9,916 host-seeking female blackflies was caught during the three sampling trips, of which 6,142 (62%) were obtained by vector collectors, 2,207 (22%) were trapped in the man-baited tents and 1,567 (16%) were caught in the cow-baited tents (Table 1). Overall, the mean hourly catches of *S. damnosum* s.l. from vector collectors (12.65 flies/person/hour; 95% CI: 11.44–13.85) were significantly higher than those from both the man-baited tents (3.41; 95% CI: 2.71–3.69), *t* = 14.97, d.f. = 456, *p* < 0.001) and the cow-baited tents (2.41; 95% CI: 2.00–2.82, *t* = 15.694, d.f. = 434, *p* < 0.001). The number of flies caught in the human-baited tents was significantly higher than that in the cow-baited tents (*t* = 3.727, d.f. = 596, p < 0.001). There were, however, no statistically significant differences between catching success by all three methods at Gyankobaa in the wet season (*F* = 0.970, d.f. = 2, p = 0.388), or Bosomase in the dry season (*F* = 0.559, d.f. = 2, p = 0.577). Only a single blackfly was caught by the electric nets, which was a male of the Beffa form of *S. soubrense* and therefore not host-seeking; no blackflies were caught in the biconical traps. However, tsetse flies and mosquitoes were caught in them, indicating that the traps were functioning as expected, but not attracting and/or catching blackflies. The final successful catching techniques used in each location for each sampling trip are summarised in Table 1.

### Blackfly identification

Of the 9,916 female blackflies collected, 212 (2%) were not *S. damnosum* s.l. These were only caught in the cow- or man-baited tents, and were subsequently identified as being *S.* (*Phoretomyia*) *berneri, S.* (*Lewisellum*) *ovazzae, S.* (*Phoretomyia*) sp.*, S.* (*Meilloniellum*) *adersi* or *S.* (*Pomeroyellum*) sp. These flies were caught at Asubende, Asukawkaw Ferry, Dodi Papase and Pillar 83 in the dry seasons and at Gyankobaa in the wet season (Additional file 1). More non-*damnosum* flies were collected in the cow-baited tents (n = 124) than in the human-baited tents (n = 88) although this difference was not statistically significant (*t* = 1.097, d.f. = 259, p = 0.274). No non-*damnosum* flies were attracted to vector collectors.

Of the 9,704 host-seeking *S. damnosum* s.l. flies collected, 8,708 (89.7%) were successfully assigned to a specific taxon (Additional file 1). The verdict of non-definitive identification in the remaining flies (n = 996) was due to a range of reasons: 1) flies too dry for morphological identification (n = 300); 2) non-successful amplification of the H3H4 gene (n = 13); 3) *S. squamosum-*negative molecular result (143 bp), but with more than one other potential species remaining from morphological identifications (n = 17); 4) molecular hybrid results (both the 143 bp and the 133 bp genes observed) (n = 61); 5) unexpected unusual molecular hybrid results (133 bp and 123 bp genes observed) (n = 4); or 6) morphological and/or molecular identifications not performed (n = 601).

Amplified DNA from subsections of all non-*S. squamosum* species (with the indel, 143 bp), the *S. squamosum* (without the indel, 133 bp) and the hybrids (133 bp and 143 bp), along with all of the unusual hybrids (123 bp and 133 bp) were sequenced. These results, combined with further sequences from ovipositing flies collected as part of the larger study will be reported elsewhere, in an attempt to elucidate how the hybrid molecular results may reflect true differences in sibling species. Any female blackflies without definitive identifications to date have been excluded from the analyses.

### *Simulium damnosum* s.l. species distribution by village, season and trapping technique

Host-seeking blackfly species distributions differed between villages, across seasons and with trapping technique. The total numbers of each sibling species collected using each technique at every location and classified according to seasons are shown in ‘Additional file 1’.

At Asubende and Agborlekame only the savannah species *S. damnosum* s.s.*/S. sirbanum* were collected. At Bosomase only forest flies were collected, with just *S. sanctipauli* found in the wet season in 2009, but with significantly different numbers of both *S. yahense* and *S. sanctipauli* found in the dry season in 2010 (χ2 = 660.8, d.f. = 1, p < 0.001). In the Volta region, at all three villages, *S. squamosum* was the dominant species, with fewer *S. damnosum* s.s.*/S. sirbanum* and *S. soubrense*. In total, three *S. yahense* were also caught in the Volta region, one at Dodi Papase and two at Pillar 83, all in 2011. In Gyankobaa, a range of forest and savannah species was collected, with *S. sanctipauli* being the dominant species, and no recorded *S. soubrense*. In the Volta region, all *S. squamosum* were C type, whilst in Gyankobaa all *S. squamosum* were E type [1]. The Djodji form of *S. sanctipauli* was not recorded at any of the sampling locations.

Four villages were successfully sampled in both seasons; Bosomase described above and Asukawkaw Ferry, where significantly more *S. soubrense* were caught in the wet season than in the dry seasons (χ2 = 421.5, d.f. = 2, p < 0.001), and Dodi Papase (χ2 = 2.9, d.f. =2, p = 0.235) and Pillar 83 (χ2 = 0.7, d.f. = 2, p = 0.403), where no statistically significant differences in sibling species distributions between wet and dry seasons were observed.

### Biting rates of sibling species of the *S. damnosum* complex

The HBR of *S. damnosum* s.l. on vector collectors ranged from zero, at a range of locations and times, to 122 bites/person/hour at Pillar 83 in the dry season of 2010 between 14:00 and 15:00 hours. The second highest biting rate of 81 bites/person/hour was also recorded at Pillar 83 at a similar time of 15:00 to 16:00 hours but in the following dry season in 2011. On both occasions the majority of blackflies were *S. squamosum*.

The highest hourly biting rates observed at each of the other villages were 29 bites/person/hour (dry season 2011, 17:00–18:00) in Asubende; 13 bites/person/hour (dry season 2010, 16:00–17:00) in Agborlekame; 64 bites/person/hour (dry season 2010, 14:00–15:00) in Asukawkaw Ferry; 41 bites/person/hour (at both dry seasons of 2010 and 2011, 14:00–15:00) in Dodi Papase; 42 bites/person/hour (wet season 2009, 14:00–15:00) in Bosomase, and 27 bites/person/hour (wet season 2009, 17:00–18:00) in Gyankobaa.

The highest hourly biting rates for each individual taxon ranged from 7 bites/person/hour for *S. yahense* at Bosomase (dry season 2010, 16:00–17:00); 8 bites/person/hour for *S. soubrense* at Asukawkaw Ferry (dry season 2010, 11:00–12:00); 29 bites/person/hour for *S. damnosum* s.s./*S. sirbanum* at Asubende (dry season 2011, 17:00–18:00); 42 bites/person/hour for *S. sanctipauli* at Bosomase (wet season 2009, 14:00–15:00), to 120 bites/person/hour for *S. squamosum* at Pillar 83 (dry season 2010, 14:00–15:00).

The lowest DBRs were recorded at Gyankobaa (dry season, 2010), where no flies were caught over 11 hours (0 bites/person/day), and Bosomase (dry season, 2011), where only 2 flies were collected over 11 hours (2 bites/person/day). No further sampling was performed at Bosomase during that collection trip/season due to this lack of blackflies, possibly attributed to pollution of the breeding sites by gold-mining activities. In contrast, the highest daily biting rate was recorded at Pillar 83 (dry season, 2010), with 353 bites/person/day. The highest mean DBRs were also at Pillar 83 (dry season 2011), with an average of 307 bites/person/day. The lowest mean daily biting rate was at Agborlekame (dry season, 2010), with 26 bites/person/day. Monthly biting rates (MBRs) are tabulated for all villages and sampling occasions by trapping method and blackfly species in Table 2.

**Table 2:** **Monthly biting rates (MBRs) by locality, season, trapping technique and sibling species of the** ***S. damnosum*** **complex**

**Region**	**Village**	**Season**	**Trapping method**	***S. damnosum*** **s.l.** ^**c**^ **total**	***S. damnosum*** **s.s** ***/S. sirbanum***	***S. soubrense*** **Beffa form**	***S. squamosum***	***S. yahense***	***S. sanctipauli***
**Brong-Ahafo**	**Asubende**	**Dry February 2011**	**V/C**	2061	2061	-	-	-	-
			**Human-Tent**	198	198	-	-	-	-
			**Cow-Tent**	238	238	-	-	-	-
	**Agborlekame**	**Dry February 2010**	**V/C**	775	775	-	-	-	-
			**Human-Tent**	32	32	-	-	-	-
			**Cow-Tent**	0	0	-	-	-	-
**Volta**	**Asukawkaw Ferry**	**Wet August 2009**	**V/C**	-	-	-	-	-	-
			**Human-Tent**	1349	0	1241	108	-	-
			**Cow-Tent**	208	0	185	23	-	-
		**Dry March 2010**	**V/C**	5777	1955	320	3502	-	-
			**Human-Tent**	1057	122	137	798	-	-
			**Cow-Tent**	951	38	198	715	-	-
		**Dry February 2011**	**V/C**	5429	1545	247	3637	-	-
			**Man-Tent**	760	131	25	605	-	-
			**Cow-Tent**	373	81	32	260	-	-
	**Dodi Papase**	**Wet August 2009**	**V/C**	-	-	-	-	-	-
			**Human-Tent**	519	7	0	512	-	-
			**Cow-Tent**	321	7	7	307	-	-
		**Dry March 2010**	**V/C**	2357	137	83	2136	-	-
			**Human-Tent**	312	0	15	297	-	-
			**Cow-Tent**	117	0	0	117	-	-
		**Dry February 2011**	**V/C**	4371	367	22	3971	-	-
			**Human-Tent**	1079	60	12	1007	-	-
			**Cow-Tent**	378	43	0	324	11	-
	**Pillar 83/Djodji**	**Wet July 2009**	**V/C**	-	-	-	-	-	-
			**Human-Tent**	15	-	-	15	-	-
			**Cow-Tent**	91	-	-	91	-	-
		**Dry March 2010**	**V/C**	7171	321	-	6776	-	-
			**Human-Tent**	494	0	0	494	-	-
			**Cow-Tent**	586	0	8	578	-	-
		**Dry February 2011**	**V/C**	9329	96	20	9167	20	-
			**Human-Tent**	2748	159	0	2589	-	-
			**Cow-Tent**	1298	152	0	1146	-	-
**Western**	**Bosomase** ^**a**^	**Wet August 2009**	**V/C**	5481	-	-	-	-	5481
			**Human-Tent**	1447	-	-	7	-	1440
			**Cow-Tent**	1613	-	-	-	-	1613
		**Dry February 2010**	**V/C**	1209	-	-	-	385	824
			**Human-Tent**	1011	-	-	-	631	380
			**Cow-Tent**	731	-	-	-	544	187
**Ashanti**	**Gyankobaa** ^**b**^	**Wet August 2009**	**V/C**	4121	247	-	486	0	3388
			**Human-Tent**	4910	33	-	9	108	4760
			**Cow-Tent**	4366	120	-	0	46	4200

Calculated monthly biting rates (MBR) of host-seeking blackflies by locality, season, trapping technique and species: ^a^Bosomase in the Dry season in 2011 for one day, V/C =2 *S. sanctipauli* =61bites/month/person; ^b^Gyankobaa in the Dry season in 2010 for 7 hrs, Cow-Tent =2 *S. damnosum s.s./S. sirbanum* =96flies/month/cow; ^c^ *S. damnosum* s.l. column equates to the total MBR for all species combined and is directly comparable with previous studies which have not split the data between species.

Monthly biting rates (MBRs) ranged from 714 bites/person/month at Agborlekame in February (all *S. damnosum* s.s./*S sirbanum*) up to 8,586 bites/person/month at Pillar 83 also in February (98.5% *S. squamosum*). Biting rates differed among villages, between seasons and by blackfly species and trapping method (Table 2). The highest monthly catching rates in the man- and cow-baited tents were 4,910 and 4,366 flies/trap/month respectively, both in Gyankobaa (wet season, 2009). Although the baited-tent catches do not correlate closely enough with the traditional OCP vector collector values to be interpreted as biting rates (Pearson correlation coefficients ranged from −0.06 to 0.5), they do offer information on the relative attraction of simuliid species towards humans or cattle. All species, except *S. damnosum* s.s.*/S. sirbanum*, exhibited a strong preference for human over cattle bait, demonstrated by both the higher overall, as well as species-specific, numbers caught in the human-baited tents (Table 2). By contrast, *S. damnosum* s.s.*/S. sirbanum* showed a preference for cattle over humans (χ^2^ = 6.91, d.f. = 1, p = 0.009). At Asukawkaw Ferry in the dry season, *S. soubrense* flies also had a slight preference for cattle over humans (χ^2^ = 6.42, d.f. = 1, p = 0.011), but this was not confirmed elsewhere for this species (χ^2^ = 1.04, d.f. = 1, p = 0.307).

### Diurnal patterns

**Figure 3:**
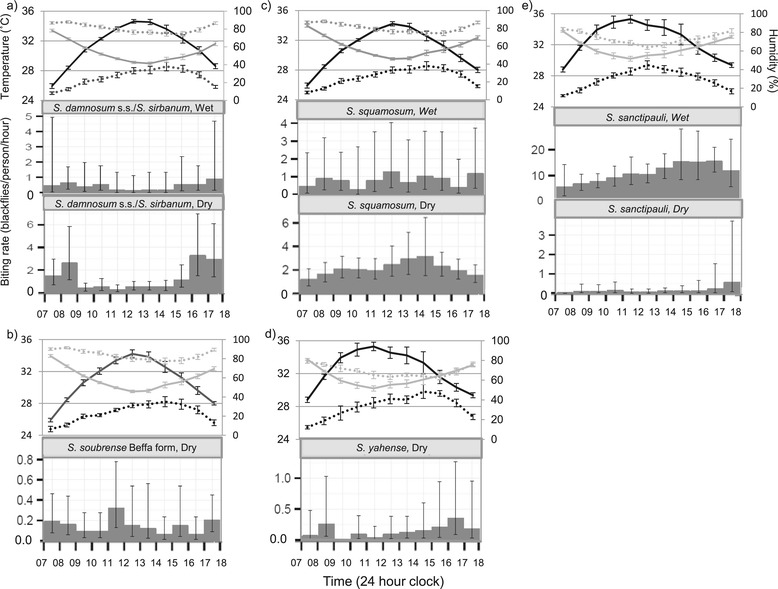
**Diurnal distribution of vector collector caught host-seeking** ***S. damnosum*** **s.l.** Daily variation of temperature (black) and humidity (grey) for dry (solid lines) and wet season (dotted lines) with temperature on the left axis (°C) and humidity on the right axis (%) are shown above the diurnal host-seeking activity for each taxon: **a)** *S. damnosum* s.s./*S. sirbanum*; **b)** *S. soubrense* Beffa form; **c)** *S. squamosum*; **d)** *S. yahense* and **e)** *S. sanctipauli*. The x-axis shows the time (hourly), and the y-axis, the mean number of blackflies caught per hour. N.B. The scales of the y-axes vary.

Hourly biting patterns varied among sibling species (Figures 3a to e). *S. damnosum* s.s.*/S. sirbanum* showed a distinct bimodal pattern, peaking in the morning between 07:00 to 09:00, and in the afternoon between 15:00 and 18:00. By contrast, *S. sanctipauli* and *S. squamosum* had unimodal distributions, with bites peaking in the afternoon. Vector-collector data are available for *S. soubrense* and *S. yahense. S. soubrense* from the dry season only. Host-seeking activity peaked between 11:00 and 15:00, and *S. yahense* showed a weak bimodal pattern, although limited fly densities hampered a more conclusive characterisation of diurnal activity for these species.

### Parous rates

**Figure 4:**
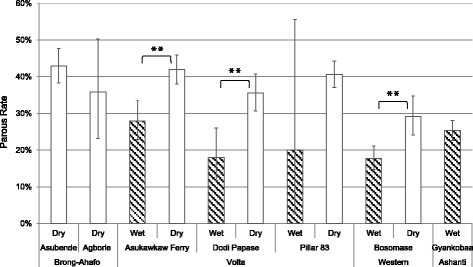
**Effect of season on parous rates of** ***S. damnosum*** **s.l.** 95% exact confidence intervals are shown. **P < 0.01.

Parity was consistently higher in the dry season than in the wet season for all sampling occasions (Figure 4, z = 11.6, p < 0.001). Overall, the mean parous rate in the dry season was 41.8% (95% CI 39.2–44.6%) compared to 18.4% (95% CI 13.7–24.2%) in the wet season. This pattern of *S. damnosum* s.l. parity was consistent in all four, and significant in three of the four, locations where both seasons were successfully sampled, with flies approximately twice as likely to be parous in the dry season (Table 3, Figure 4). The large confidence intervals at Agborlekame and Pillar 83 (in the wet season) are due to low blackfly numbers with only 53 and 10 blackflies collected, respectively.

**Table 3:** **Parous rates of sibling species of the** ***S. damnosum*** **complex by locality, season and trapping technique**

**Region**	**Village**	**Season**	**Trapping method**	***S. damnosum*** **s.l**	***S. damnosum*** **s.s.** ***/S. sirbanum***	***S. soubrense*** **Beffa form**	***S. squamosum***	***S. yahense***	***S. sanctipauli***
**Brong-Ahafo**	**Asubende**	**Dry February 2011**	**V/C**	42.5 (37.6-47.7)	42.5 (37.6-47.7)	-	-	-	-
			**Human-Tent**	32.4 (19.1-49.2)	32.4 (19.1-49.2)	-	-	-	-
			**Cow-Tent**	54.8 (40.0-68.8)	54.8 (40.0-68.8)	-	-	-	-
	**Agborlekame**	**Dry February 2010**	**V/C**	35.3 (23.6-49.0)	35.3 (23.6-49.0)	-	-	-	-
			**Human-Tent**	50.0 (9.1-90.6)	50.0 (9.1-90.6)	-	-	-	-
			**Cow-Tent**	-	-	-	-	-	-
**Volta**	**Asukawkaw Ferry**	**Wet August 2009**	**V/C -**	-	-	-	-	-	-
			**Human-Tent**	28.8 (23.5-34.7)	-	30.9 (25.3-37.1)	5 (0.9-23.6)	-	-
			**Cow-Tent**	22.2 (11.7-38.1)	-	18.8 (8.9-35.3)	50.0 (15.0-85.0)	-	-
		**Dry March 2010**	**V/C**	45.2 (38.5-52.0)	43.5 (25.6-63.2)	35.3 (21.5-52.1)	47.7 (39.8-55.6)	-	-
			**Human-Tent**	42.3 (33.9-51.1)	37.5 (18.5-61.4	47.1 (26.2-69.0)	42.2 (32.5-52.5)	-	-
			**Cow-Tent**	47.0 (38.2-56.0)	100 (56.6-100)	64.0 (44.5-79.8)	40.2 (30.6-50.7)	-	-
		**Dry February 2011**	**V/C**	43.5 (30.2-57.8)	100 (43.9-100)	42.9 (15.8-75.0)	38.9 (24.8-55.1)	-	-
			**Human-Tent**	34.4 (25.6-44.5)	25.0 (10.2-49.5)	100 (43.9-100)	38.4 (28.1-49.8)	-	-
			**Cow-Tent**	27.7 (16.9-41.8)	60.0 (31.3-83.2)	0 (0–49.0)	21.9 (11.0-38.8)	-	-
	**Dodi Papase**	**Wet August 2009**	**V/C**	-	-	-	-	-	-
			**Human-Tent**	18.4 (11.3-28.6)	0 (0–79.4)	-	18.7 (11.5-28.9)	-	-
			**Cow-Tent**	17.4 (9.1-30.7)	100 (20.7-100)	ND	15.8 (7.8-28.8)	-	-
		**Dry March 2010**	**V/C**	36.3 (30.0-43.2)	ND	80.0 (49.0-94.3)	34.0 (27.7-41.0)	-	-
			**Human-Tent**	26.8 (16.0-41.9)	-	50.0 (9.5-90.6)	25.6 (14.6-41.1)	-	-
			**Cow-Tent**	46.2 (23.2-70.9)	-	-	46.2 (23.2-70.9)	-	-
		**Dry February 2011**	**V/C**	ND	ND	ND	ND	-	-
			**Man-Tent**	34.3 (24.3-46.0)	25.0 (4.6-69.9)	ND	34.9 (24.5-46.9)	-	-
			**Cow-Tent**	40.0 (25.6-56.4)	25.0 (4.6-69.9)	-	42.2 (27.4-60.8)		-
	**Pillar 83/Djodji**	**Wet July 2009**	**V/C**	-	-	-	-	-	-
			**Man-Tent**	0 (0–65.8)	-	-	0 (0–79.4)	-	-
			**Cow-Tent**	22.2 (6.3-54.7)	-	-	22.2 (6.3-54.7)	-	-
		**Dry March 2010**	**V/C**	46.9 (40.2-53.7)	100 (34.2-100)	-	46.4 (39.7-53.2)	-	-
			**Human-Tent**	41.9 (30.5-54.3)	-	-	41.9 (30.5-54.3)	-	-
			**Cow-Tent**	42.7 (32.1-54.0)	-	0 (0–79.4)	43.2 (32.6-54.6)	-	-
		**Dry February 2011**	**V/C**	47.4 (37.6-57.3)	33.3 (6.2-79.2)	100 (20.7-100)	48.3 (38.2-58.6)	0 (0–65.8)	-
			**Human-Tent**	29.3 (22.7-36.8)	0 (0–21.5)	-	32.2 (25.1-40.2)	-	-
			**Cow-Tent**	37.5 (29.8-45.9)	0 (0–17.6)	-	43.2 (34.6-52.2)	-	-
**Western**	**Bosomase**	**Wet August 2009**	**V/C**	18.4 (13.7-24.2)	-	-	-	-	18.4 (13.7-24.2)
			**Man-Tent**	15.4 (10.9-21.3)	-	-	ND	-	15.4 (10.9-21.3)
			**Cow-Tent**	19.3 (14.3-25.4)	-	-	-	-	19.3 (14.3-25.4)
		**Dry February 2010**	**V/C**	29.4 (21.0-39.3)	-	-	-	31.3 (10.0-45.3)	27.3 (16.4-41.9)
			**Man-Tent**	26.2 (19.4-34.3)	-	-	-	23.2 (15.4-33.4)	31.3 (20.0-45.3)
			**Cow-Tent**	34.2 (24.5-45.4)	-	-	-	36.2 (25.1-49.1)	26.3 (11.8-48.8)
**Ashanti**	**Gyankobaa**	**Wet August 2009**	**V/C**	ND	ND	-	ND	-	ND
			**Human-Tent**	24.7 (21.4-28.4)	75.0 (30.1-95.4)	-	100 (20.7-100)	76.9 (49.7-91.8)	23.0 (19.7-26.7)
			**Cow-Tent**	26.1 (28.5-43.7)	77.8 (45.3-93.7)	-	-	60.0 (23.1-88.2)	31.0 (26.8-35.6)

Summary of parous rates in percentages (95% exact confidence intervals) of host-seeking blackflies by locality, season, trapping technique and species. Some parous rates are not available as none of the flies from that sample, or of that specific species were dissected for parity: Not Dissected (ND).

### Diurnal distributions of parous rates

**Figure 5:**
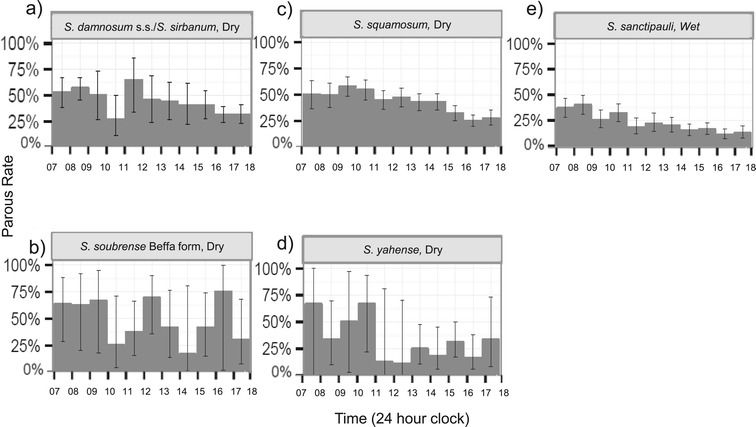
**Diurnal distribution of the proportion of parous flies for each sibling species.** The x-axis shows the time (hourly), and the y-axis, the parous rate per hour (%). Parous rates calculated from all trapping methods combined. The number of flies of each species dissected per season varied and are presented for each species for the season with the larger sample sizes and greater confidence in the data: **a)** *S. damnosum* s.s./*S. sirbanum*; dry season n = 603, **b)** *S. soubrense* Beffa form dry season n = 260, **c)** *S. squamosum* dry season n = 1,506; **d)** *S. yahense* dry season n = 191; and **e)** *S. sanctipauli* wet season n = 1,563. Graphs for all species for both seasons are shown in ‘Additional file 2’.

The number of flies of each taxon dissected for parity per season varied: *S. damnosum* s.s./*S. sirbanum*; dry season n = 603, wet season n = 15: *S. squamosum* dry season n = 1506, wet season n = 155: *S. sanctipauli* dry season n = 111, wet season n = 1563: *S. soubrense* dry season n = 106, wet season n = 260: *S. yahense* dry season n = 191, wet season n = 18. The estimated parity (Figure 5a to e) of both *S. squamosum* in the dry season (Figure 5a) and *S. sanctipauli* in the wet season (Figure 5e) was higher in the morning than in the evening and, moreover, the accompanying confidence intervals do not overlap. This indicates a statistically significant difference in parity between the morning and the evening for these two species. Although limited sample sizes precluded a more conclusive characterisation of diurnal parity for other taxa, the highest parous rates occurred in the morning for four of the five species, particularly between 08:00 and 09:00 for *S. sanctipauli,* between 09:00 and 10:00 for *S. squamosum,* between 11:00 and 12:00 for *S. damnosum* s.s.*/S. sirbanum,* and between 10:00 and 11:00 for *S. yahense.* There was no clear pattern for *S. soubrense* Beffa form*.*

### Monthly parous biting rates of sibling species of the S. damnosum complex

Some MPBRs were not calculated for vector collector-caught flies, as when fly catches were greater than approximately 300 flies per day for the tent-caught flies, the vector-collector caught flies were not dissected in the field and therefore parity was not assessed. Where MPBR results were available, they varied among species, location and between seasons (Table 4). The overall MPBR in the dry season was 1,823, statistically significantly higher than that in the wet season, 1,028 (*t* = 6.679, d.f. = 212, p < 0.001). The highest MBPR was observed at Pillar 83 (dry season, 2011), with an average of 4,419 parous bites/person/month. The lowest MBPR was recorded at Agborlekame (dry season, 2010), with an average of 252 parous bites/person/month. In general, *S. squamosum* type C contributed the most to the MBPR, through high levels in all three Volta region villages. *S. sanctipauli* contributed the most to MBPR in the southern forested villages of Bosomase and Gyankobaa. Vector collector-derived MBPR were statistically significantly higher in the wet season in Bosomase than in the dry season both for all blackflies (*t* = 3.477, d.f. = 61, p = 0.001) and for *S. sanctipauli* alone (*t* = 4.922, d.f. = 49.8, p < 0.001) due to the high biting rates in the wet season. The highest MPBRs for the *S. damnosum* s.s.*/S. sirbanum* savannah species were recorded at Asubende, with 806 parous bites/person/month in the dry season. No data are available for the wet season from the Brong-Ahafo region, but *S. damnosum* s.s.*/S. sirbanum* also contributed the most to the MPBR at Asukawkaw Ferry (dry season, 2011), with 1,422 parous bites/person/month.

**Table 4:** **Monthly parous biting rates (MPBRs) by village, season, trapping technique and sibling species of the** ***S. damnosum*** **complex**

**Region**	**Village**	**Season**	**Trapping method**	**Total** ***S. damnosum*** **s.l**	***S. damnosum*** **s.s.** ***/S. sirbanum***	***S. soubrense*** **Beffa form**	***S. squamosum***	***S. yahense***	***S. sanctipauli***
**Brong-Ahafo**	**Asubende**	**Dry February 2011**	**V/C**	877	877	-	-	-	-
			**Human-Tent**	64	64	-	-	-	-
			**Cow-Tent**	130	130	-	-	-	-
	**Agborlekame**	**Dry February 2010**	**V/C**	274	274	-	-	-	-
			**Human-Tent**	16	16	-	-	-	-
			**Cow-Tent**	0	0	-	-	-	-
**Volta**	**Asukawkaw Ferry**	**Wet August 2009**	**V/C -**	-	-	-	-	-	-
			**Human-Tent**	389	0	383	5	-	-
			**Cow-Tent**	98	0	35	12	-	-
		**Dry March 2010**	**V/C**	2608	850	113	1669	-	-
			**Human-Tent**	447	48	64	337	-	-
			**Cow-Tent**	447	38	127	288	-	-
		**Dry February 2011**	**V/C**	2360	1545	106	1414	-	-
			**Human-Tent**	262	33	25	232	-	-
			**Cow-Tent**	103	49	0	57	-	-
	**Dodi Papase**	**Wet August 2009**	**V/C**	-	-	-	-	-	-
			**Human-Tent**	96	0	0	96	-	-
			**Cow-Tent**	56	7	0	48	-	-
		**Dry March 2010**	**V/C**	856	ND	66	727	-	-
			**Man-Tent**	84	0	8	76	-	-
			**Cow-Tent**	54	0	0	54	-	-
		**Dry February 2011**	**V/C**	ND	ND	ND	ND	-	-
			**Man-Tent**	370	15	0	351	-	-
			**Cow-Tent**	151	11	0	140		-
	**Pillar 83/Djodji**	**Wet July 2009**	**V/C**	-	-	-	-	-	-
			**Human-Tent**	0	-	-	0	-	-
			**Cow-Tent**	20	-	-	7	-	-
		**Dry March 2010**	**V/C**	3362	321	-	3142	-	-
			**Human-Tent**	207	0	0	207	-	-
			**Cow-Tent**	250	0	0	250	-	-
		**Dry February 2011**	**V/C**	4419	32	20	4429	0	-
			**Man-Tent**	805	0	0	833	-	-
			**Cow-Tent**	487	0	0	495	-	-
**Western**	**Bosomase**	**Wet August 2009**	**V/C**	1006	-	-	-	-	1006
			**Human-Tent**	223	-	-	ND	-	223
			**Cow-Tent**	311	-	-	-	-	311
		**Dry February 2010**	**V/C**	355	-	-	-	120	225
			**Human-Tent**	264	-	-	-	146	119
			**Cow-Tent**	250	-	-	-	197	49
**Ashanti**	**Gyankobaa**	**Wet August 2009**	**V/C**	ND	ND	-	ND	0	ND
			**Human-Tent**	1213	25	-	9	83	1095
			**Cow-Tent**	1139	93	-	0	28	1042

Estimates of monthly parous biting rates (MPBR) of host-seeking blackflies by locality, season, trapping technique and species. Some MBPRs are not available as none of the flies from that sample, or of that specific species were dissected for parity: Not Dissected (ND).

## Discussion

We demonstrated the existence of significant spatial and temporal variation in species distribution, host-seeking behaviour, daily biting activity, and parous rates. As selected African countries move towards onchocerciasis elimination goals [60], criteria for the certification of interrupted transmission, as for the Americas, including guidelines for entomological evaluation of the impact of community ivermectin distribution and other interventions, are needed [61,62]. These guidelines specifically include determination of biting and infection rates by timed systematic vector collections in localities reflecting human exposure to, and vector abundance during, peak times of parasite transmission (both by season and time of day) to maximise the detection of infected/infective flies [61,63]. We investigated the biting and parous rates of six simuliid onchocerciasis vectors in Ghana, one of the countries in which vector control operations, under the OCP umbrella, started in 1975 and mass ivermectin distribution, trialled in 1987, was subsequently rolled out.

We confirmed the expectation (from cytotaxonomic larval identification at corresponding breeding sites) [1], that only *S. damnosum* s.s.*/S. sirbanum* are found biting in the savannah villages of Asubende and Agborlekame, whilst a range of taxa was caught (as host-seeking adult females) in the Volta region villages, with *S. squamosum* being the predominant anthropophagic species. In Gyankobaa, a village in the forested Ashanti Region, a mixed composition of species was documented. This highlights the identification complexities which can only be resolved by combined morphological and molecular criteria, as well as the transmission complexities that may arise from exposure to ensembles of vector species with different vector competences and vectorial capacities. Although the majority of host-seeking blackflies collected at Gyankobaa were *S. sanctipauli* (a forest species), nearly 10% were *S. damnosum* s.s.*/S. sirbanum* (savannah species). The remainder were *S. squamosum* and *S. yahense* (the latter predominantly a forest species). Only *S. soubrense* s.s. was lacking from this ensemble during our study, but this species was represented by the Beffa form at three sites in the Volta Region. The moderate presence of savannah flies in this forested region may reflect a potential extension of their range, possibly due to deforestation in southern Ghana [64], although they do not appear to have statistically significantly increased in frequency in this area according to analyses presented in Post *et al.* [1]. At Bosomase, in the southern forest region, no adult host-seeking savannah flies were recorded, with only *S. sanctipauli* (in the 2009 wet season) and both *S. sanctipauli* and *S. yahense* (the latter at moderate densities) caught in the 2010 dry season. Due to the absence of flies at Bosomase in the 2011 dry season and only one wet season trip, we cannot conclude whether the presence of *S. yahense* in 2010, but not 2009, is due to yearly variation, differences between seasons, or a recent invasion of *S. yahense* into an area previously only occupied by *S. sanctipauli*. However, the latter is supported by ovipositing flies collected by IT in the 2006 dry season (data not shown), which consisted of only *S. sanctipauli* with over 3,350 flies identified. In addition, no *S. yahense* larvae or pupae were collected at Bosomase at any historical sampling times [1], indicating that these flies may be feeding near the River Pra but not breeding in the river. Typically, *S. yahense* prefers smaller, cooler rivers [65,66] and probably breeds in nearby tributaries. Analyses of ovipositing flies collected as part of the larger entomological study, strongly support this suggestion, with no *S. yahense* observed, but with 415 *S. sanctipauli* collected, 354 of them at the same time as the host-seeking *S. yahense* in 2010. Finally, the Djodji form of *S. sanctipauli* was not recorded in any of our locations, including the 2,845 flies collected at Pillar 83 (Djodji), confirming the report of its elimination [45].

Crosskey [67] indicated that *S. damnosum* s.l. biting levels were more stable in forest than in savannah areas, and this assertion is supported here, with bimodal peaks (in the morning and early evening) of host-seeking *S. damnosum* s.s.*/S. sirbanum* in the savannah localities of Asubende and Agborlekame, that were much less pronounced for these species in all three Volta Region villages or in Gyankobaa. The remainder of the sibling species showed less distinct diurnal patterns in biting and parity, with an overall decreasing trend throughout the day, similar to trends documented for forest-dwelling blackflies in Amazonian onchocerciasis foci [33]. These observations are likely to be explained by the higher temperatures and lower humidities around midday that prevail in dry savannah areas. Daily temperature and humidity fluctuations are less pronounced in forested areas, potentially explaining the more uniform biting patterns of the forest species.

In contrast to our findings, Adeleke and co-workers observed bimodal parous biting peaks in one rainforest area, but early evening unimodal peaks, in a second rainforest area and a savannah area of Nigeria [68]. This study, however, did not identify species and the reported bimodal patterns could either be interpreted as true bimodality specific to the study areas, or different taxa biting at different diurnal times. In addition, only parous biting rates were presented, not separating biting from parous rates and their relative contribution to the recorded peaks. Other studies on diurnal patterns of parous rates in the savannah in Africa have also reported morning peaks [33] while others have reported midday peaks [36,56]. Grillet *et al.* [33] discussed that hourly patterns of parity in host-seeking simuliid populations may relate to breeding site proximity, as blackfly peak oviposition activity takes place at dusk or dawn [67]. Morning parous peaks indicate greater breeding site proximity, with peaks later in the day suggesting that host-seeking flies have travelled longer distances. Le Berre (1966) has shown that host-seeking dispersal differs between parous and nulliparous, with parous rates declining with distance from oviposition sites [36].

Our investigations into the biting and parity patterns of the sibling species of the *S. damnosum* complex permit, together with *O. volvulus* infection rates reported elsewhere, a more precise identification of the seasons and diurnal times during which *O. volvulus* transmission is potentially highest [69]. This provides useful information for the design of entomological evaluation protocols for the monitoring of onchocerciasis control and elimination programmes [31,63,70,71]. Our findings indicate that collecting during mornings in the dry season would increase the chances of sampling parous flies and, therefore, the likelihood of *O. volvulus* infected flies. Higher parous rates in the dry season were consistently shown in *S. damnosum* s.l. studies conducted by Le Berre [36] and Le Berre *et al*. [72] in Burkina Faso; by Philippon [73] in Burkina Faso, Côte d’Ivoire, Democratic Republic of Congo and Mali, and by Adeleke *et al*. [68] and Adewale *et al*. [74] in Nigeria, in agreement with the results presented here. In addition, comparison of our parous rates in February for Asubende (42.5%) and Agborlekame (35.0%) to parous rates observed by OCP prior to vector control indicates very similar levels, at 40.7% and 38.7% respectively. In Djodji, historical studies which separated the taxa, showed parous rates of 56.9% in February and 32.2% in March for *S. squamosum* [47], in comparison to our findings of 48.3% and 46.4% for February and March, respectively. Differences may either be due to natural annual variation, their lower sample sizes and/or changes over time with species composition and/or vector control. Parous rates of ~40% recorded from a Nigerian savannah focus [68] are also similar to those observed in these savannah villages of Asubende and Agborlekame. However, in the forest villages we investigated, host-seeking flies had lower parous rates than those reported for Nigerian forest regions, potentially due to different species compositions.

The MPBR is one of the most important epidemiological measures in entomological monitoring, encompassing both biting density and parity status. Although parous rates were twice as high in the dry season than the wet season, the resulting MPBRs reflected the higher MBRs observed during the wet season (in contrast with the results of [74]). In general, transmission is expected to be greater in the wet season, with some sites drying out in the dry season. However, at perennial sites transmission may peak in the dry season [75]. Our net values indicated higher mean MPBRs in the dry season due to the high parous rates, whilst other studies show similar MPBR results but for different reasons. For example, higher dry season MPBRs have been shown to be due to exposed breeding sites which were flooded in the wet season [36,76]. These complexities highlight that, for entomological monitoring and evaluation, simply choosing periods of high vector density/biting rates will not necessarily maximise the potential for assessing changes in *O. volvulus* transmission. This has important programmatic implications. Firstly, not collecting during the periods of higher vector abundance will require more intensive sampling effort to obtain enough flies to power statistically meaningful assessments of changes in infection rates [31]. Secondly, not assessing parity status in the field (usually not performed in programmatic contexts due to the specialised and labour-intensive requirements) may lead to erroneously low infection rates (as nullipars will harbour no infection). Statistically sound considerations need to be taken into account when designing entomological evaluation procedures and using pool-screening methods to be able to handle the demands of very large sample sizes [43,77]. Also, a thorough understanding of seasonal patterns of transmission will become increasingly important as programmes based on community-directed treatment with ivermectin (CDTI) endeavour towards elimination. The effectiveness (and cost effectiveness) of treatment has been shown to be sensitive to the timing of peak transmission in relation to the timing of mass ivermectin distribution [Turner HC, Walker M, Attah SK, Opoku NO, Awadzi K, Kuesel AC, Basáñez MG: The potential impact of moxidectin on onchocerciasis elimination in Africa: an economic evaluation based on the Phase II clinical trial data. Submitted].

In comparison to historical OCP MBR data, our February value for Asubende of 1,897bites/person/month is similar to the February mean of the 8-year sampling (1978–1985) conducted prior to vector control (2,012 ± 594 bites/person/month), but higher than the February mean in the final 8 years of larviciding (1999–2002) (580 ± 154 bites/person/month). This indicates that after cessation of vector control, blackfly populations have returned to their original levels and ecological conditions propitious to transmission may favour the persistence of infection when *O. volvulus* populations remain despite prolonged ivermectin treatment. Indeed, our vector infection results (presented elsewhere) suggest that *O. volvulus* transmission may continue in/around Asubende, despite intensified (biannual) ivermectin distribution, and a related entomological study has documented active transmission in Agborlekame [Veriegh FBD, Basáñez MG, Armoo S, Cheke RA, Walker M, Boakye DA, Wilson MD, Taylor M, Osei-Ateweneboana MY: Continuing transmission of *Onchocerca volvulus* in and near zones with poor responses to ivermectin: implications for onchocerciasis elimination in Ghana. In *preparation*]. OCP data for Agborlekame, collected from 1975 to 1988—throughout which time vector control was occurring—indicates, not surprisingly, a lower February mean (311 ± 212 bites/person/month) than our February biting rate (775 bites/person/month) post vector control.

In the Volta region, some historical OCP MBR data exist, prior to the initiation of larvicidal operations (1979 to 1986). For Dodi Papase, the MBRs over these years were 4,051 ± 709 in February and 2,832 ± 465 in March. These values compare well with our MBRs of 4,024 and 2,402 for the same months, respectively, indicating that in the absence of vector control simuliid populations have reverted to their original equilibrium levels. In contrast, even though MBRs at Pillar 83 (Djodji) were the highest observed throughout our study—at over 7,000 bites/person/month for February and March—these are lower than historical levels (1979 to 1986), with mean MBRs of 12,907 ± 1,720 and 11,389 ± 1,622 bites/person/month for February and March respectively, despite the fact that there were vector control trials conducted in 1981 [43]. This very high historical vector density was almost certainly due to the presence of the highly anthropophilic Djodji form of *S. sanctipauli*, which is now extinct [45].

### Limitations

A limitation of our study was that accurate comparisons between seasons were difficult given the lack of vector collector samples from the first four villages visited in the 2009 wet season trip, and the sporadic success of sampling at later times in the dry seasons. When it became apparent that fly catches in the tents were not truly representative of vector density, standard OCP vector collection methods were implemented. The baited tents worked with equal success for overall fly catches, and highlighted the strong anthropophilic host-seeking behaviour of most species, with the exception of *S. damnosum* s.s.*/S. sirbanum*. When using human odour or human bait behind the electric-net traps, or the biconical tsetse traps, no host-seeking female blackflies were caught. This may be because olfactory cues were not great enough, or because blackflies can sense the presence of objects and are able to fly around them, without contacting electric wires or biconical trap entrances. Although further research into blackfly trapping methods is warranted and ongoing [78], we did not obtain any evidence that either of the traps tested here may be suitable for large-scale blackfly collection to replace vector collectors. This is in contrast to other studies reporting the capture of 481 blackflies per day [53] or even higher in an unwashed, baited biconical trap of up to 2,123 flies in one day, with *S. yahense* appearing to be less likely to enter the traps than *S. sanctipauli* [54].

## Conclusions

We demonstrated substantial spatial and temporal variation in the distribution, host-seeking activity, daily biting patterns and parous rates of sibling species of the *S. damnosum* complex of onchocerciasis vectors in southern Ghana. MBRs tended to be higher in the wet season, whilst parity rates were significantly higher in the dry season, resulting in MPBRs being highest overall in the dry season. In savannah regions, exposure to parous flies (potentially carrying *O. volvulus*) reached up to 800 parous bites/person/month in the dry season by *S. damnosum* s.s.*/ S. sirbanum*, with MBRs as high as those documented prior to vector control, indicating that in the absence of antivectorial measures, ecological conditions favourable to transmission exist. These may contribute to the persistence of infection when local parasite elimination has not been achieved, or contribute to its recrudescence if infection persists in contiguous areas, both important considerations for the elimination efforts that African countries, including Ghana, have embarked upon. In the Volta region, *S. squamosum* contributed the most to the MPBRs, with over 4,000 parous bites/person/month in the dry season at Pillar 83/Djodji. In the southern forested regions, *S. sanctipauli* remains the greatest contributor to the risk of *O. volvulus* exposure. Our results indicate that for transmission dynamics models to be useful in examining the feasibility of onchocerciasis elimination across African foci, the vector component of such models would need to be modified to take into account the diversity of species that contribute to *O. volvulus* transmission on their own or in combination at sites with mixed species composition and vectorial capacities. According to their vector competence for *O. volvulus*, the contribution of different species to transmission may vary as microfilarial loads decrease due to disparities in density-dependent parasite uptake and vector survival [79]. Besides, the susceptibility of different species to add-on interventions, such as focal vector control, will have to be tested if complementary tools are to be deployed to accelerate progress towards onchocerciasis elimination.

## Additional files

Additional file 1:
**Summary of host-seeking blackflies caught, by locality, season, trapping technique and species.**
Additional file 2:
**Graphs of the parous rates of each species for both dry and wet seasons.**

## Abbreviations

CDTI: Community-directed treatment with ivermectin: the current control strategy for reducing onchocerciasis transmission, infection load and morbidity
DBR: Daily biting rate: the number of vector bites received per host in a day
HBR: Hourly biting rate: the number of vector bites received per host in an hour
MBR: Monthly biting rate: the number of vector bites received per host in a month
MPBR: Monthly parous biting rate: the number of bites by parous flies received per host in a month
MTP: Monthly transmission potential: the number of L3 larvae to which a host is exposed in a month
OCP: Onchocerciasis control programme in West Africa – run in Ghana from 1974 until 2002
PR: Parous rate: the proportion of adult female flies who are parous
s.l.: *Sensu lato*
s.s.: *Sensu stricto*
WHO: World Health Organization
