# Supplementary material for: Onchocerciasis transmission in Ghana: biting and parous rates of host-seeking sibling species of the Simulium damnosum complex
Source: Parasit Vectors. 2014 Nov 21;7:511. doi: 10.1186/s13071-014-0511-9 (PMC4247625; doi:10.1186/s13071-014-0511-9)
Supplement: Additional file 1: — Summary of host-seeking blackflies caught, by locality, season, trapping technique and species. [file 13071_2014_511_MOESM1_ESM.pdf]

Additional file 1: Summary of host-seeking blackflies caught, by locality, season, trapping technique and species

Region	Village	Season	Humidity (Mean (Range))	Temp (Mean (Range))	Trapping Method	Hours	Total	Total *S. damnosum* s.l.	*S. damnosum s.s.* /*S. sirbanum*	*S. soubrense* Beffa form	*S. squamosum*[a,b]	*S. yahense*	*S. sanctipauli*	Non-damnosum	Unidentified[c]
Brong-Ahafo	Asubende	Dry February 2011	35.7% (13-81)	32.6 °C (21.8-37.0)	V/C	62hrs	382	382	382	-	-	-	-	0	-
					Human-Tent	59hrs	70	35	35	-	-	-	-	35	-
					Cow-Tent	59hrs	112	42	42	-	-	-	-	70	-
	Agborlekame	Dry February 2010	46.9% (32-66)	34.4°C (28.8-40.2)	V/C	22hrs	51	51	51	-	-	-	-	-	-
					Human-Tent	21hrs	2	2	2	-	-	-	-	-	-
					Cow-Tent	14hrs	0	0	0	-	-	-	-	-	-
Volta	Asukawkaw Ferry	Wet August 2009	80.9% (44-99)	27.3°C (24.0-34.9)	V/C	-	-	-	-	-	-	-	-	-	-
					Human -Tent	62hrs	250	250	0	230	20	-	-	-	-
					Cow-Tent	58hrs	36	36	0	32	4	-	-	-	-
		Dry March 2010	66.4% (45-99)	30.4°C (23.9-36.1)	V/C	45hrs	777	777	227	37	407	-	-	-	44M 62D
					Human -Tent	44hrs	139	139	16	18	105	-	-	-	-
					Cow-Tent	44hrs	125	125	5	26	94	-	-	-	-
		Dry February 2011	57.3% (34-99)	30.7°C (23.1-36.0)	V/C	44hrs	714	714	175	28	412	-	-	0	28M 71D
					Human -Tent	44hrs	108	100	16	3	74	-	-	8	7D
					Cow-Tent	43hrs	58	48	10	4	32	-	-	10	2D
	Dodi Papase	Wet August 2009	93.1% (78-99)	25.3°C (22.7-27.7)	V/C	-	-	-	-	-	-	-	-	-	-
					Human -Tent	49hrs	76	76	1	0	75	-	-	-	-
					Cow-Tent	49hrs	47	47	1	1	45	-	-	-	-
		Dry March 2010	59.2% (43-99)	32.0°C (25.5-36.0)	V/C	44hrs	310	310	18	11	280	-	-	-	1M
					Human-Tent	44hrs	41	41	0	2	39	-	-		
					Cow-Tent	40hrs	14	14	0	0	13	-	-	-	1D
		Dry February 2011	56.7% (35-99)	30.3°C (22.3-36.3)	V/C	31hrs	405	405	34	2	357	-	-	0	12D
					Human -Tent	31hrs	105	100	5	1	84	-	-	5	10M
					Cow-Tent	31hrs	36	35	4	0	30	1	-	1	-
	Pillar 83 /Djodji	Wet July 2009	75.7% (41-98)	28.3°C (23.9-38.2)	V/C	-	-	-	-	-	-	-	-	-	-
					Human -Tent	44hrs	2	2	-	-	1	-	-	-	1D
					Cow-Tent	44hrs	12	12	-	-	10	-	-	-	2D
		Dry March 2010	67.0% (37-99)	29.6°C (23.9-39.9)	V/C	44hrs	943	943	26	-	549	-	-	-	9M, 359NA
					Human -Tent	44hrs	66	65	0	0	62	-	-	1	3D
					Cow-Tent	44hrs	77	77	0	1	75	-	-	-	1D
		Dry February 2011	50.8% (12-97)	32.6°C (22.0-38.8)	V/C	42hrs	1171	1171	12	2	910	2	-	0	3M 242NA
					Human -Tent	42hrs	374	345	20	0	325	-	-	29	-
					Cow-Tent	41hrs	200	159	18	0	136	-	-	41	5D
Western	Bosomase[d]	Wet August 2009	78.0% (49-99)	27.1°C (23.7-33.3)	V/C	42hrs	688	688	-	-	-	-	688	-	-
					Human -Tent	46hrs	199	199	-	-	1	-	198	-	-
					Cow-Tent	45hrs	217	217	-	-	-	-	211	-	6D
		Dry February 2010	67.7% (39-85)	32.5°C (27.5-39.6)	V/C	44hrs	159	159	-	-	-	49	105	-	5D
					Human -Tent	44hrs	134	134	-	-	-	83	50	-	1D
					Cow-Tent	38hrs	83	83	-	-	-	61	21	-	1D
Ashanti	Gyankobaa[e]	Wet August 2009	69.1% (50-89)	28.0°C (24.5-32.7)	V/C	44hrs	542	542	35	-	63	0	439	0	5D
					Human -Tent	43hrs	641	631	4	-	1	13	572	10	41D
					Cow-Tent	42hrs	550	548	13	-	0	5	456	2	74D
**Totals**					**V/C**	**464hrs**	**6142**	**6142**	**960**	**80**	**2978**	**51**	**1232**	**0**	**841**
					**Human-Tent**	**617hrs**	**2207**	**2119**	**99**	**254**	**787**	**96**	**820**	**88**	**63**
					**Cow-Tent**	**592hrs**	**1567**	**1443**	**93**	**64**	**439**	**67**	**688**	**124**	**92**

[a]All *S. squamosum* from the Volta region were type C.

[b]All *S. squamosum* from Gyankobaa were type E. The single *S. squamosum* found in Bosomase was not identified to cytoform.

[c]The unidentified *S. damnosum* s.l. flies were so classified either because they were too desiccated for successful morphological identification (D) or the molecular results could not successfully identify a specific species (M). In addition, a subsection of V/C caught flies from two samples were not identified to species as they were donated to another study (NA).

[d]In Bosomase in 2011, due to low fly intensities only two V/C *S. sanctipauli* flies were caught over one day, no further sampling was performed.

[e]In Gyankobaa in the dry season in 2010 due to low fly intensities, no V/C or Human-Tent caught flies were caught and only two *S. damnosum s.s. /S. sirbanum* were caught in the Cow-Tent over 7 hours, no further sampling was performed.
