# Supplementary figures and images for: Onchocerciasis transmission in Ghana: biting and parous rates of host-seeking sibling species of the Simulium damnosum complex
Source: Parasit Vectors. 2014 Nov 21;7:511. doi: 10.1186/s13071-014-0511-9 (PMC4247625; doi:10.1186/s13071-014-0511-9)

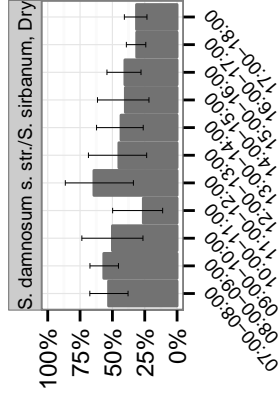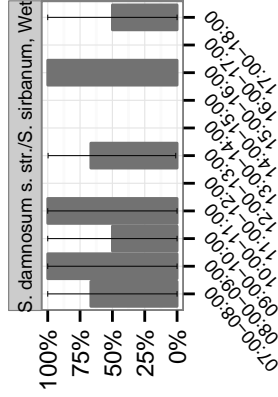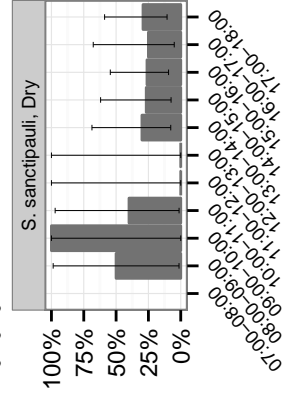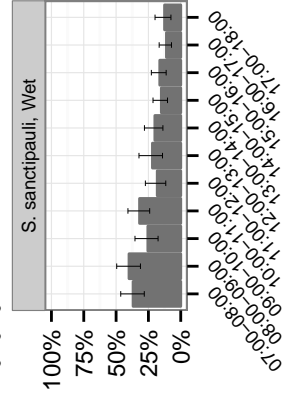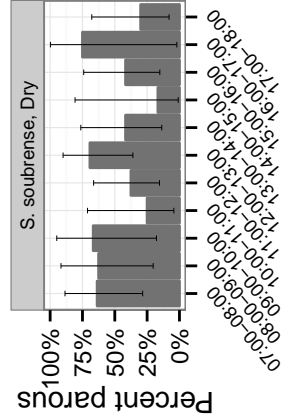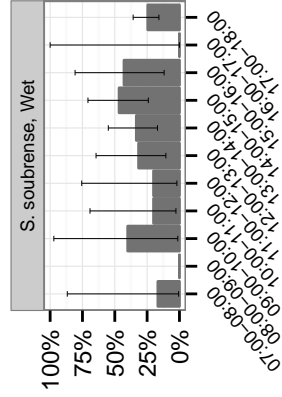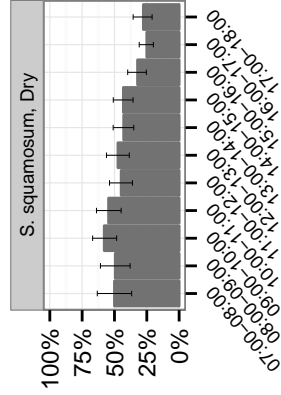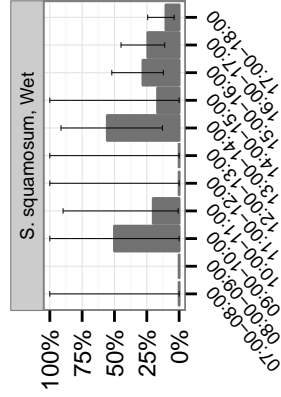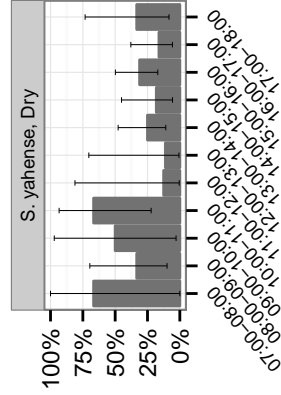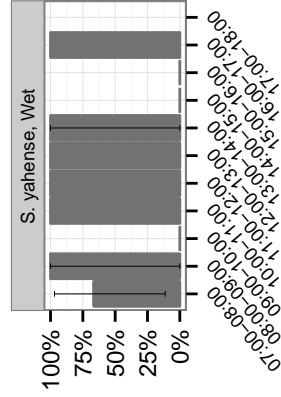

Time

Supplement: Additional file 2: — Graphs of the parous rates of each species for both dry and wet seasons. [file 13071_2014_511_MOESM2_ESM.pdf]
